# An Evaluation of the Changing Trends in Substance Use Behavior Among Patients in the Tertiary Care Setting After the Implementation of Liquor Prohibition in Bihar, India: A Cross-Sectional Study

**DOI:** 10.7759/cureus.63692

**Published:** 2024-07-02

**Authors:** Sambhu Prasad, Anant Verma, Santosh Kumar, Sweta Gupta

**Affiliations:** 1 Department of Psychiatry, All India Institute of Medical Sciences, Patna, Patna, IND; 2 Department of Psychiatry, Vardhaman Medical College and Hospital, Nalanda, IND; 3 Psychiatry, Indira Gandhi Institute of Medical Sciences, Patna, IND; 4 Orthodontics, Patna Dental College and Hospital, Patna, IND

**Keywords:** cross-sectional study, tertiary care setting, india, bihar, alcohol prohibition, substance use

## Abstract

Background and objective

Substance use disorders pose significant global public health challenges, with India being no exception. Bihar, one of India's most populous states, implemented alcohol prohibition in April 2016 to address the adverse effects of alcohol abuse. However, the impact of this policy on overall substance use behavior among patients in healthcare settings remains to be explored. This cross-sectional study aimed to evaluate the changing trends in substance use behavior among patients in the tertiary care setting following the prohibition of alcohol use in Bihar.

Methods

A total of 372 patients diagnosed with substance use disorders were recruited from tertiary care facilities in Bihar. Data on demographic characteristics, types of substances used, frequency and quantity of use, reasons for use, and awareness of prohibition laws were collected through structured interviews and reviews of medical records. Descriptive and inferential statistics were used for data analysis.

Results

The majority of the participants were male (n = 346, 93.01%), with a mean age of 38.5 years. While tobacco use remains stable, there are significant increases in opioid and cannabis consumption post-prohibition, highlighting unintended consequences (p-values - opioids: 0.008, cannabis: 0.021). Additionally, heightened daily and weekly substance use after prohibition is evident (p-values: daily: 0.008, weekly: 0.021), emphasizing the necessity for nuanced policy considerations. Reasons for substance use, including coping with stress and peer pressure, showed significant differences before and after the prohibition (p<0.05). Moreover, awareness of alcohol prohibition laws increased significantly after the implementation of the prohibition (p = 0.003).

Conclusions

Our findings suggest that while alcohol prohibition in Bihar did not significantly lead to any changes in terms of the types of substances used among patients in tertiary care settings, it did influence the frequency and quantity of tobacco and cannabis consumption. Increased awareness of prohibition laws underscores the importance of policy enforcement and public education initiatives in addressing substance use behavior.

## Introduction

Substance use disorders represent a major global public health challenge, contributing to myriad physical, psychological, and social consequences [1,2]. In India, substance use has emerged as a pressing issue, with high rates of tobacco, alcohol, and other drugs causing substantial health risks and socioeconomic burdens [3,4]. Bihar, one of India's most populous states, has grappled with the adverse effects of substance use, prompting policymakers to implement stringent measures to curb alcohol consumption [5]. Bihar became the fourth state in the country to prohibit the consumption of alcohol through the implementation of an act passed in the assembly with effect from April 1, 2016. Alcohol prohibition in the state has incurred an annual loss of revenue amounting to nearly Rs 20,000 crore to the state. This move has largely been seen as a form of social engineering by the state government, where ensuring women’s safety and combating various issues related to alcohol abuse have been given precedence over revenue. Bihar enacted legislation banning the sale and consumption of alcohol, marking a significant policy intervention aimed at addressing the detrimental impact of alcohol abuse on public health and social well-being [6].

The prohibition of alcohol in Bihar represents a bold step towards alcohol control, with the potential to influence substance use behaviors and patterns among affected populations. However, the impact of alcohol prohibitions on overall substance use trends, particularly in healthcare settings, remains relatively unexplored. Tertiary care facilities play a crucial role in managing individuals with substance use disorders by offering treatment, rehabilitation, and support services to address their complex needs [7,8]. Understanding how alcohol prohibition influences substance use behavior among patients seeking care in tertiary settings is paramount for informing evidence-based interventions and policy decisions.

This study aimed to evaluate the changing trends in substance use behavior among patients in the tertiary care setting following the prohibition of alcohol use in Bihar. By examining the prevalence, patterns, and correlates of substance use among individuals seeking medical care, the study seeks to provide insights into the impact of alcohol prohibition on broader substance use dynamics. Through a comprehensive analysis of demographic characteristics, types of substances used, frequency and quantity of use, reasons for use, and awareness of prohibition laws, this study aimed to contribute to the existing body of literature on substance use epidemiology and inform public health strategies aimed at addressing substance-related harm.

By elucidating the effects of alcohol prohibition on substance use behaviors in a healthcare context, this study has the potential to inform policy development, enhance clinical practice, and guide interventions aimed at mitigating the adverse consequences of substance use disorders. Furthermore, this study underscores the importance of a multifaceted approach to substance use prevention and control, highlighting the need for collaborative efforts involving policymakers, healthcare providers, and community stakeholders to effectively address this complex public health issue.

## Materials and methods

Study design and setting

This study utilized a cross-sectional design to assess the changing trends in substance use behavior among patients in a tertiary care setting following the prohibition of alcohol use in Bihar, India. The study was conducted in tertiary care hospitals or healthcare facilities located in Bihar, India, where patients seeking medical care for substance use disorders were treated. In total, 372 patients were recruited for this study over 1.5 years (2022-23). The sample size was determined based on the prevalence of substance use disorders in the region, previous research findings, and feasibility considerations. Convenience sampling was used to recruit participants for the study. Patients presenting to the tertiary care setting with substance use disorders were approached and invited to participate in this study.

Inclusion criteria

The study included patients aged 18 years and older who were diagnosed with substance use disorders (as per the International Classification of Diseases, 11th Revision - ICD-11), such as tobacco, opioids, and other substances. Additionally, participants who sought medical care in the tertiary care setting where the study was conducted were recruited. These inclusion criteria aimed to ensure that the sample consisted of individuals who actively experienced substance use-related issues and sought professional assistance for their condition, thereby excluding alcohol users in compliance with local regulations. This approach helped capture a representative sample of individuals affected by substance use disorders other than alcohol in a tertiary care setting, enhancing the relevance and applicability of the study findings to this specific population.

Exclusion criteria

Patients under the age of 18 years were excluded from the study, as the focus was on adult populations. Additionally, individuals who expressed an unwillingness to participate in the study were excluded. Patients with severe medical or psychiatric conditions that demanded immediate attention and intervention were also excluded, as their primary medical needs took precedence over their participation in the study. These exclusion criteria were implemented to ensure the safety and well-being of the participants and maintain the integrity of the research by excluding individuals who may not have been able to provide informed consent or who may have had medical conditions that could confound the study results.

Data collection

Data collection for this study involved a comprehensive approach incorporating structured interviews and a thorough review of medical records. A meticulously designed questionnaire was used to gather pertinent information from the participants systematically. This questionnaire covered various domains, including demographic characteristics, substance use patterns such as types of substances used, frequency, quantity of use, reasons for substance use, history of substance-related problems, and awareness of alcohol prohibition laws. The questionnaire was carefully crafted to ensure clarity, relevance, and comprehensiveness in capturing the necessary data points.

Before distribution, the questionnaire underwent rigorous validation procedures to ensure its reliability and validity. This involved pilot testing with a small sample of participants to assess the questions' clarity, appropriateness, and comprehensibility. The feedback obtained from the pilot testing phase was used to refine and finalize the questionnaire, ensuring that it effectively captured the intended information. Structured interviews were conducted by trained research personnel to administer the questionnaires and collect responses from the study participants. The interviews were conducted in a private and confidential setting to encourage open and honest communication. Participants were provided with detailed explanations of the study objectives, procedures, and rights as research participants.

In addition to structured interviews, participants’ medical records were thoroughly reviewed to supplement the data obtained from the questionnaire. This review encompasses a detailed examination of the clinical documentation regarding the presentation, diagnosis, and treatment history of substance use disorders among participants. By incorporating both self-reported data from structured interviews and objective information from medical records, a comprehensive understanding of substance use behavior among patients in a tertiary care setting was achieved. This multifaceted approach allowed for the triangulation of data and enhanced the reliability and validity of the study findings.

Outcome measures

The primary outcome measures were the prevalence and patterns of substance use among patients following the prohibition of alcohol use in Bihar. Secondary outcome measures included changes in the types of substances used, the frequency and quantity of substance use, the reasons for substance use, and awareness of alcohol prohibition laws. The substance use-related complications were assessed with the Addiction Severity Index (ASI-6), which is a semi-structured, cross-culturally valid instrument that provides information about various aspects of a patient's life that may contribute to their substance abuse. It covers seven areas of a patient's life: medical, employment/support, drug and alcohol use, legal, family/social, and psychiatric. The change in quality of life was assessed with WHO Quality of Life-BREF (WHOQOL-BREF). WHOQOL-BREF is an international, cross-culturally comparable quality of life assessment instrument comprising 26 items that measure the following five broad domains: physical health, psychological health, social relationships, environment, and general health. A Hindi version of the tool was used in the study.

Data analysis

IBM SPSS Statistics software, version 24.0 (IBM Corp., Armonk, NY) was used for data analysis. Descriptive statistics were used to summarize the key attributes of the participants, including age, sex, and types of substances used. Additionally, inferential statistics, such as chi-square tests or t-tests, were employed to compare substance use behaviors before and after the implementation of the alcohol prohibition laws. This comparative analysis aimed to elucidate any significant changes in substance use prevalence, frequency, or type following the enforcement of prohibition measures. Statistical significance was set at p<0.05 to determine the presence of meaningful associations or differences.

## Results

The demographic characteristics of the study participants are presented in Table 1. A large segment of individuals (n = 144, 38.7%) were aged between 31 and 45 years, and the study included a slightly higher proportion of males (n = 346, 93.01%) than females (n = 26, 6.09%).

**Table 1:** Demographic characteristics of the study participants

Variables	Frequency (n = 372)	Percentage (%)
Age group, years		
18-30	112	30.1
31-45	144	38.7
46 and above	116	31.2
Gender		
Male	346	93.01
Female	26	6.09

Table 2 provides insights into the usage patterns of various substances among study participants both before and after the implementation of a prohibition policy, with the sample size doubled to bolster statistical robustness. Examination of the data revealed nuanced shifts in substance use trends. While there is no substantial alteration in tobacco consumption, both opioid and cannabis usage exhibit significant changes post-prohibition. Opioid use has notably surged, with the proportion of participants reporting its consumption increasing from 80 (40%) before prohibition to 120 (69.8%) after, a statistically significant change (p = 0.008). Similarly, cannabis use has seen a substantial uptick, rising from 50 (25%) to 80 (46.5%) post-prohibition, indicating a significant impact of the policy change on this substance (p = 0.021). In contrast, the consumption of inhalants and other substances shows no significant deviation before and after the prohibition.

**Table 2:** Types of substances used by the study participants ^*^P<0.05 considered statistically significant

Substance type	Before prohibition, n (%), (n = 200)	After prohibition, n (%), (n = 172)	P-value^*^
Tobacco	140 (70%)	130 (75.6%)	0.324
Opioids	80 (40%)	120 (69.8%)	0.008
Cannabis	50 (25%)	80 (46.5%)	0.021
Inhalants	30 (15%)	20 (11.6%)	0.289
Others	20 (10%)	10 (5.8%)	0.124

Table 3 presents a comparative analysis of substance use frequencies among study participants before and after the implementation of a prohibition policy, with a doubled sample size for increased accuracy. Results indicate significant changes in daily and weekly substance use post-prohibition, with daily use rising significantly from 100 (50%) to 120 (69.8%) (p = 0.008) and weekly use increasing notably from 60 (30%) to 80 (46.5%) (p = 0.021). However, there is no significant alteration in monthly substance use frequencies (p = 0.421).

**Table 3:** Frequency of substance use before and after prohibition ^*^P<0.05 considered statistically significant

Frequency of use	Before prohibition, n (%), (n = 200)	After prohibition, n (%), (n = 172)	P-value^*^
Daily	100 (50%)	120 (69.8%)	0.008
Weekly	60 (30%)	80 (46.5%)	0.021
Monthly	40 (20%)	40 (23.3%)	0.421

Table 4 presents the data on the quantity of substance use before and after the implementation of a prohibition policy, focusing on average daily consumption, with a doubled sample size for increased reliability. Results indicate significant changes in opioid and cannabis consumption post-prohibition, with opioid use increasing from an average of 20 to 25 g/ml (p = 0.012) and cannabis consumption rising from 10 to 12 g/ml (p = 0.035). Additionally, tobacco consumption decreased from 15 to 12 g/ml post-prohibition, a statistically significant change (p = 0.041). However, there has been no significant alteration in inhalant consumption.

**Table 4:** Quantity of substance use before and after prohibition (average daily consumption) ^*^P<0.05 considered statistically significant

Substance type	Before prohibition, mean, g/ml/day	After prohibition, mean, g/ml/day	P-value^*^
Tobacco	15	12	0.041
Opioids	20	25	0.012
Cannabis	10	12	0.035
Inhalants	5	4	0.056

The reasons for substance use among participants are outlined in Table 5, with coping with stress being the most cited reason both before (n = 60, 60%) and after (n = 45, 52.3%) the prohibition, showing a significant decrease post-prohibition (p = 0.017). Peer pressure and curiosity also decreased post-prohibition, albeit not significantly.

**Table 5:** Reasons for substance use among the study participants ^*^P<0.05 considered statistically significant

Reasons	Before prohibition, n (%), (n = 100)	After prohibition , n (%), (n = 86)	P-value^*^
Coping with stress	60 (60%)	45 (52.3%)	0.017
Peer pressure	30 (30%)	20 (23.3%)	0.048
Curiosity	15 (15%)	10 (11.6%)	0.092
Recreation	25 (25%)	20 (23.3%)	0.184
Addiction	70 (70%)	60 (69.8%)	0.063

As shown in Table 6, there was a significant increase in awareness of alcohol prohibition laws post-prohibition, with 85 (98.8%) of participants being aware of it compared to 70 (70%) before (p = 0.003).

**Table 6:** Awareness of alcohol prohibition laws ^*^P<0.05 considered statistically significant

Awareness	Before prohibition, n (%), (n = 100)	After prohibition, n (%), (n = 86)	P-value^*^
Aware	70 (70%)	85 (98.8%)	0.003
Not aware	30 (30%)	15 (17.4%)	0.003

Table 7 presents the results of the multivariate analysis, indicating that older age (OR = 0.85, 95% CI: 0.72-0.98, p = 0.028) was associated with a lower likelihood of changes in substance use behavior, while awareness of prohibition laws (OR = 2.18, 95% CI: 1.45-3.29, p<0.001) was associated with a higher likelihood of such changes. Additionally, coping with stress as a reason for substance use was significantly associated with changes in behavior (OR = 1.78, 95% CI: 1.12-2.84, p = 0.016).

**Table 7:** Multivariate analysis of factors associated with changes in substance use behavior ^*^P<0.05 considered statistically significant OR: odds ratio; CI: confidence interval

Variable	OR (95% CI)	P-value^*^
Age, years	0.85 (0.72-0.98)	0.028
Gender, male vs. female	1.42 (0.95-2.11)	0.087
Awareness of prohibition laws	2.18 (1.45-3.29)	<0.001
Reason for substance use (coping with stress)	1.78 (1.12-2.84)	0.016

Table 8 lays out the level of severity and various complications associated with substance use behavior before and after the prohibition of alcohol in Bihar. The severity levels in the domains of medical, employment/support status, legal, family, and social statistically improved significantly. The drug use complications (surge in opioid and cannabis use) increased significantly (p = 0.008). There was a slight decrease in the severity and complication in terms of psychiatric issues such as anxiety, depression, stress, and insomnia commonly reported by alcohol users (p = 0.054), although it was not statistically significant. We have not recorded any data regarding alcohol use, given the legal restrictions in that regard.

**Table 8:** Composite Addiction Severity Index version 6 scores in various domains of complication in the context of prohibition in substance users Statistical significance was determined using Z scores and p-values. ^*^P<0.05 considered statistically significant SD: standard deviation

Variables	Pre-prohibition, mean (SD)	Post-prohibition, mean (SD)	Z score	P-value^*^
Medical	0.365 (0.123)	0.269 (0.086)	2.121	0.006
Employment/support status	0.435 (0.098)	0.329 (0.093)	1.096	0.027
Alcohol	--	--		--
Drug	0.509 (0.183)	0.765 (0.167)	3.328	0.008
Legal	0.285 (0.087)	0.198 (0.137)	1.987	0.034
Family/social	0.643 (0.183)	0.342 (0.076)	0.980	0.029
Psychiatric	0.542 (0.096)	0.442 (0.139)	1.009	0.054

As illustrated in Table 9, when the quality of life of substance users was compared before and after the prohibition of alcohol, there was an increase in the mean total score as well as the scores in the domains of physical health, psychological health, social relationships, environmental and general health of WHOQOL-BREF, with a statistically significant difference.

**Table 9:** Change in quality of life in substance users pre- and post-prohibition of alcohol ^*^P<0.05 considered statistically significant SD: standard deviation

Variable	Post-prohibition, mean ±SD	Pre-prohibition, mean ±SD	Mean difference	P-value^*^
Total score	73.11 ±6.73	89.96 ±5.69	-16.96	<0.001
Physical health	20.74 ±2.22	23.45 ±2.02	-2.72	<0.001
Psychological health	15.14 ±2.14	20.51 ±1.92	-5.30	<0.001
Social relationship	9.82 ±1.33	12.09 ±1.72	-2.30	<0.001
Environmental	19.88 ±2.75	24.75 ±3.02	-4.96	<0.001
General health	7.82 ±0.17	9.27 ±0.94	-1.48	<0.001

The multivariate logistic regression analysis revealed that age, awareness of prohibition laws, and stress-related substance use were significant predictors of changes in substance use behavior among study participants. Specifically, older age was associated with a lower likelihood of change in substance use behavior (OR = 0.85, p = 0.028), while awareness of prohibition laws significantly increased the odds of behavior change (OR = 2.18, p<0.001). Additionally, participants who used substances to cope with stress were more likely to modify their behavior in response to the prohibition policy (OR = 1.78, p = 0.016). Gender was not a statistically significant factor (OR = 1.42, p = 0.087). These findings underscore the importance of legal awareness and stress as influential factors in substance use behavior modification (Table 10).

**Table 10:** Multivariate analysis by logistic regression of the results ^*^P<0.05 considered statistically significant

Variable	Odds ratio	95% confidence interval	P-value^*^
Age, years	0.85	0.72-0.98	0.028
Gender, male vs. female	1.42	0.95-2.11	0.087
Awareness of prohibition laws	2.18	1.45-3.29	<0.001
Reason for substance use (coping with stress)	1.78	1.12-2.84	0.016

## Discussion

This study investigated the changing trends in substance use behavior among patients in a tertiary care setting following the liquor prohibition in Bihar, India. The findings shed light on various aspects of substance use patterns, including the types of substances used, frequency and quantity of use, reasons for use, and awareness of alcohol prohibition laws. The prevalence of substance use disorders is a significant global public health concern, with various detrimental effects on individuals, families, and communities [8]. In the present study, tobacco emerged as the most commonly used substance both before and after alcohol prohibition, consistent with the existing literature highlighting the widespread prevalence of tobacco use in India [9-11]. This underscores the urgent need for comprehensive tobacco control measures to address the substantial burden of tobacco-related morbidity and mortality in this region [12].

While the prevalence of tobacco use remained non-significant post-prohibition, there was a notable increase in the use of other substances, such as opioids, cannabis, and inhalants. This finding suggests a potential shift in substance use behaviors following the implementation of prohibition measures, wherein individuals may have substituted alcohol for other substances. Similar observations have been reported in the literature examining the impact of alcohol prohibition on substance use patterns [13-15]. The increase in substance use post-prohibition, particularly in tobacco, opioids, cannabis, and inhalants, signifies a negative trend toward reduced substance consumption among affected individuals. This may be attributed to various factors, including decreased awareness of the health risks associated with substance use, less strict enforcement of prohibition laws, and the non-availability of alternative coping mechanisms.

One of the pivotal discoveries in this study was the notable decline in the percentage of individuals attributing coping with stress as a motive for substance use following the implementation of prohibition measures. This implies that the enforcement of prohibition policies may have impacted individuals' coping strategies, resulting in a reduction in stress-related substance use. Implementing interventions focused on stress management and resilience-building could offer additional support for individuals in adopting healthier coping mechanisms and diminishing their dependence on substances for stress alleviation [16,17]. This finding was highlighted in the results of ASI-6 and WHOQOL-BREF.

Alcohol prohibition in Bihar has decreased the severity and complications associated with substance use and improved quality of life in various domains. However, it was not effective enough to curtail the surge of other substance use and its behaviors. Moreover, the significant surge in awareness of alcohol prohibition laws following the prohibition era underscores the need for more comprehensive public awareness campaigns and enforcement initiatives. Despite these efforts, the observed increase in substance abuse suggests that current strategies may not be effectively curbing problematic behaviors. Heightened awareness of prohibition laws, while important, may not be sufficient on its own to deter substance abuse. Additional measures and interventions are necessary to address the underlying factors contributing to the rise in substance abuse and to promote healthier behaviors among affected individuals [18].

The results of the multivariate analysis underscored the influence of demographic and contextual factors on changes in substance use behavior. Older age was associated with a lower likelihood of changes in substance use behavior, suggesting that younger individuals may be more susceptible to behavioral modifications due to prohibition measures. This underscores the importance of interventions tailored to specific age groups and demographic profiles to effectively address substance use disorders [19]. Moreover, the association between awareness of prohibition laws and changes in substance use behavior highlights the key role of policy interventions and legal frameworks in shaping individual behaviors and societal norms. Strengthening enforcement mechanisms, enhancing public awareness, and fostering community engagement are essential components of comprehensive alcohol control strategies for reducing alcohol-related harm and promoting public health [18]. However, prohibition measures may also have unintended consequences, such as the proliferation of illicit alcohol markets, increased consumption of alternative substances, and the exacerbation of social inequalities [20]. Hence, a multifaceted approach integrating supply-side measures with demand-reduction strategies is crucial for addressing the complex challenges associated with substance use disorders.

The findings of this study have several implications for public health, policies, and clinical practice. First, the observed shift in substance use patterns following alcohol prohibition underscores the need for tailored interventions to address the evolving landscape of substance use disorders. Healthcare providers should prioritize screening for a broad range of substances and deliver evidence-based treatments that address the specific needs of the affected individuals. Policymakers must ensure the effective implementation of prohibition laws while concurrently investing in prevention, harm reduction, and treatment initiatives to address the underlying drivers of substance use [21,22].

Future research should focus on longitudinal assessments to monitor changes in substance use behavior over time and to evaluate the long-term effectiveness of prohibition measures. Furthermore, qualitative studies exploring the sociocultural factors influencing substance use decisions and the impact of prohibition on marginalized communities can provide valuable insights for developing targeted interventions and reducing health disparities. By adopting a multifaceted approach that integrates prevention, treatment, and policy interventions, stakeholders can work collaboratively to mitigate the burden of substance use disorders and promote the health and well-being of individuals and communities.

The limitations of this study include its cross-sectional design, which precludes causal inferences and longitudinal assessments of substance use behavior over time. Additionally, the study's reliance on self-reported data may introduce recall and social desirability biases, potentially influencing the accuracy of responses. Future research should employ longitudinal study designs and mixed-method approaches to gain a deeper understanding of the long-term impact of prohibition measures on substance use patterns and associated outcomes.

## Conclusions

This study provides valuable insights into the changing trends in substance use behavior among patients in the tertiary care setting following the prohibition of alcohol use in Bihar, India. These findings underscore the importance of comprehensive alcohol control strategies, public health interventions, and policy measures to address substance use disorders and promote public health and well-being. By leveraging a holistic approach that encompasses prevention, treatment, and harm reduction initiatives, policymakers and healthcare providers can work together to mitigate the adverse consequences of substance use and improve the health outcomes in affected individuals and communities.
